# Cross-Species Analysis of Milk Extracellular Vesicles Reveals a Conserved Innate Core and a Divergent, Human-Specific Adaptive Immune Fraction

**DOI:** 10.3390/ijms27188142

**Published:** 2026-09-12

**Authors:** Eleni Papakonstantinou, George P. Chrousos, Dimitrios Vlachakis

**Affiliations:** 1 Laboratory of Genetics, Department of Biotechnology, Agricultural University of Athens, 11855 Athens, Greece; eleni@aua.gr; 2 University Research Institute of Maternal and Child Health and Precision Medicine, Medical School, National and Kapodistrian University of Athens, 11527 Athens, Greece; chrousos@gmail.com; 3 Algorithms and Bioinformatics Group, Informatics Department, Faculty of Natural, Mathematical & Engineering Sciences, King’s College, Strand Campus, London WC2R 2LS, UK

**Keywords:** milk extracellular vesicles, milk exosomes, inflammation markers, innate immunity, cross-species conservation, exosomal microRNA, epigenetic regulation, TLR4/NF-κB, miR-148a, maternal–infant immune signaling

## Abstract

Milk-derived extracellular vesicles (EVs) carry proteins and microRNAs that mediate immune communication between mother and offspring, yet which inflammation-related cargo is conserved—and which is species specific—across mammals remains poorly defined. We assembled MetaMilkDB, a provenance-tracked database integrating proteomic, transcriptomic, metabolomic and pathway-level evidence for milk-EVs across four species (*Homo sapiens*, *Bos taurus*, *Capra hircus*, *Ovis aries*), combining curated studies with in-house EV proteomics (265,009 measurements). Of 4958 EV protein families, 570 (11.5%) formed a conserved core containing five inflammation markers (HP, LTF, MUC1, MUC15, TLR2), four re-detected in our in-house proteomes. This core was an innate scaffold and was not itself enriched for inflammation; instead, inflammation cargo concentrated in the species-specific fraction, overwhelmingly in human milk (45/966 vs. 6/1222 shared; odds ratio 9.9; q = 1.6 × 10^−10^), forming a secretory-immunoglobulin and complement module absent from ruminant cargo. A parallel layer of inflammation-annotated EV-microRNAs showed heterogeneous conservation across species and converged on TLR/NF-κB-related immune regulation. Milk-EV immunity is organized on two axes—a conserved innate scaffold shared across species and a divergent, largely human adaptive-immune fraction—clarifying which cargo is a candidate cross-species biomarker and which may underlie species-specific immune transfer.

## 1. Introduction

Extracellular vesicles (EVs) are nanometre-scale, membrane-bound particles secreted by virtually all cell types, including the secretory epithelium of the lactating mammary gland. They mediate intercellular communication by transferring bioactive cargo—proteins, lipids, mRNAs, long non-coding RNAs and microRNAs—between cells, organs and even organisms [1,2,3]. Because this cargo carries both genetic and epigenetic information, EVs can act horizontally, delivering functional transcripts and gene-regulatory microRNAs that reshape the expression program of recipient cells, and they are increasingly recognized as vehicles through which a mother can durably shape the developing physiology and immune repertoire of her offspring [4,5,6]. Milk-EV microRNAs in particular fine-tune recipient-cell gene expression post-transcriptionally, providing an epigenetic dimension to maternal–infant signaling that complements the vesicles’ protein cargo [6,7].

Milk is an exceptionally rich and continuously renewed source of EVs. Milk-EVs survive gastrointestinal transit, are taken up by intestinal and immune cells [8,9], and deliver functional microRNAs and immunomodulatory proteins to the neonate [1,4]. Their stability and low immunogenicity have also made them attractive as natural drug-delivery scaffolds [10]. A central, unresolved question is the extent to which the molecular content of milk-EVs is conserved across mammalian species. Conservation matters on two fronts: evolutionarily conserved cargo is more likely to be functionally essential, and cargo shared between humans and dairy species (cow, goat, sheep) underpins the safety and translational rationale for using animal milk-EVs in human nutrition and therapeutics [11].

Despite a growing body of single-species proteomic and transcriptomic surveys [2,12,13], cross-species integration has been hampered by heterogeneous identifiers, inconsistent reporting of vesicle purity, and the frequent conflation of computationally predicted annotations with experimentally observed ones. Inflammation-related cargo is a particularly attractive focus: milk-EVs are repeatedly reported to modulate the neonatal immune system and to attenuate inflammatory gut disease [14,15,16], and a marker set conserved across humans and dairy species would be directly useful both as a cross-species inflammation biomarker panel—for example, for mammary inflammation (mastitis)—and as the molecular basis for the safe nutritional use of animal milk-EVs. To address this, we built MetaMilkDB, a harmonized database that integrates milk and milk-EV omics across four target species under a strict provenance and data-honesty policy, combining 78 curated public studies with in-house EV proteomics generated within the project. Here we use MetaMilkDB to resolve milk-EV immunity along two complementary axes. We first define the conserved protein core shared by all four species and the compact innate-immune signature within it—HP, LTF, MUC1, MUC15 and TLR2—corroborated by our in-house proteomes. We then show that inflammation cargo is not concentrated in this conserved core but in the species-specific fraction, most dramatically in human milk, where it forms a secretory-immunoglobulin and complement module with no ruminant counterpart. Conservation and divergence thus encode different kinds of immunity—a shared innate scaffold and a species-tuned adaptive layer—with direct consequences for biomarker design and for the use of animal milk-EVs in human nutrition.

## 2. Results

### 2.1. A Harmonised Four-Species Milk-EV Resource

MetaMilkDB integrates evidence from 78 curated public studies together with in-house EV proteomics, spanning proteomics, transcriptomics, metabolomics, milk composition, EV characterization and curated pathway annotation. The in-house layer comprised 12 datasets (data_sources flagged in_house) contributing 265,009 protein measurements across four species (*Homo sapiens*, *Bos taurus*, *Capra hircus*, *Ovis aries*), and 18 independently characterized human milk-EV preparations with measured RNA and protein yields (Section 2.8). After identifier resolution, the database contained 20,019 distinct curated features, of which 19,299 (96.4%) were resolved to a named or canonical accession and 720 were retained with an explicit needs-lookup status rather than being discarded or guessed. The large majority of features were derived from the milk-EV compartment (16,103 features; 80.4%) rather than whole milk (3916 features), and 18,300 features were assigned to the four core target species, with a smaller extended set (1707 features) from non-target species (buffalo, donkey) retained for comparison but excluded from the conserved-core analysis. A complete breakdown of the curated public studies—species, level of omic analysis, EV-isolation method, vesicle characterization and full citation—is provided in Supplementary Table S1.

### 2.2. Cross-Species Conservation of the Milk-EV Proteome

**Figure 1:**
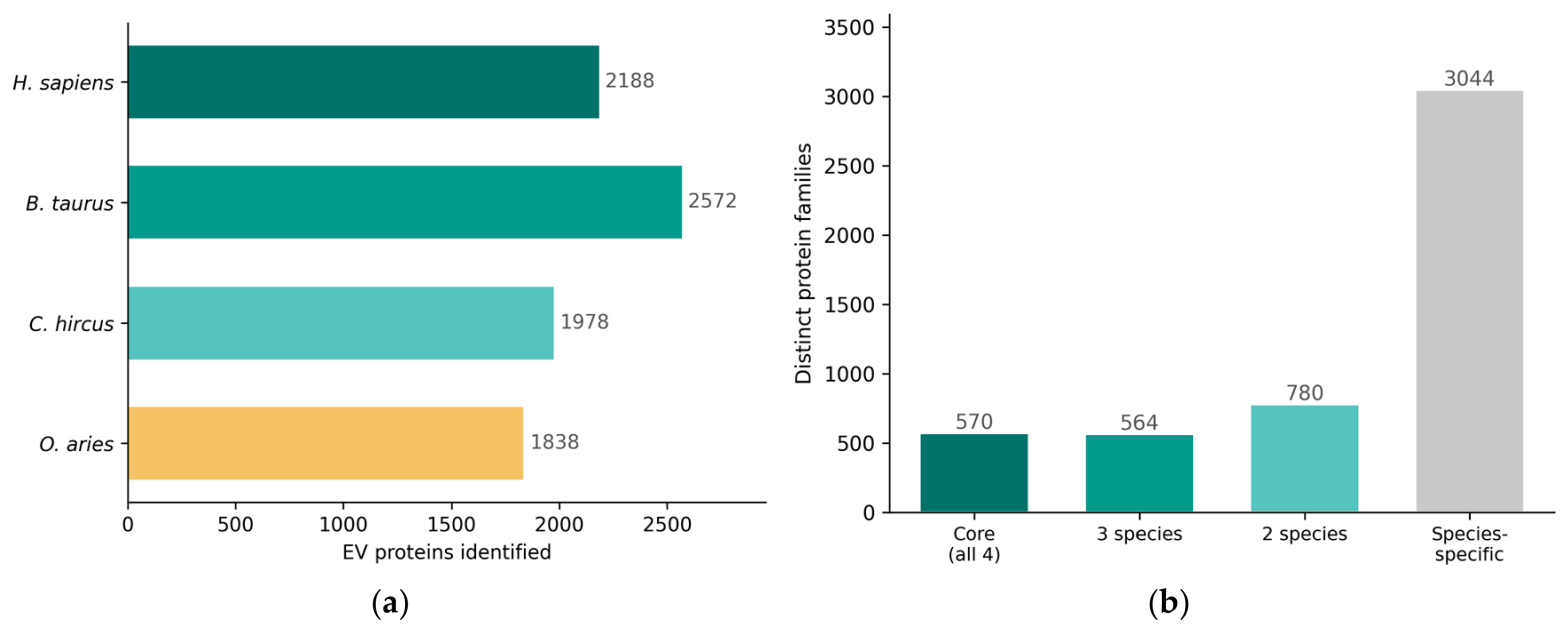
Milk-EV proteome depth and conservation. (**a**) Distinct EV protein families identified per species. (**b**) Partition of the 4958-family union by the number of species in which each family is detected, defining a 570-family conserved core.

Restricting the analysis to EV-compartment proteins from the four core species and grouping by canonical protein family yielded 4958 distinct families. The number of families identified per species was comparable across the four (*Bos taurus*, 2572; *Homo sapiens*, 2188; *Capra hircus*, 1978; *Ovis aries*, 1838), consistent with broadly similar EV-proteome depth despite differing study counts. Partitioning families by the number of species in which they were detected revealed a clear conserved core: 570 protein families (11.5% of the union) were present in all four species, 564 in exactly three, 780 in exactly two, and 3044 were species specific (Figure 1). Thus, 1134 families (22.9%) were shared by at least three species, indicating a substantial conserved backbone superimposed on a larger, species-variable periphery.

Because conservation is scored as presence across species, the size of the conserved core is sensitive to sampling depth, which is greatest for the most-studied (bovine) arm. To control for this, all four species were downsampled to the depth of the shallowest proteome (1838 families), and the analysis was repeated over 200 iterations. The four-species core decreased from 570 to a mean of 319 families (56%; 95% interval 299–335), and each of the five inflammation markers was retained in the rarefied core in 52–59% of iterations (Supplementary Figure S1). A conserved fraction and a recurrent enrichment of the five markers, therefore, persist after equalizing sampling effort, indicating that the conserved core is robust to sampling-depth bias, although its absolute size is best interpreted as an upper estimate.

### 2.3. A Conserved Five-Marker Inflammation Proteomic Signature

We next focused on the central question: which conserved-core proteins carry explicit inflammation- or innate-immune evidence. Applying the database inflammation flag—set only where a source study explicitly annotates an inflammatory or immune role, never inferred—exactly five of the 570 conserved-core families were positive: haptoglobin (HP), lactotransferrin/lactoferrin (LTF), the mucins MUC1 and MUC15, and Toll-like receptor 2 (TLR2). Each is annotated with a distinct, source-grounded rationale (Table 1): LTF as an antimicrobial pathogen-protection factor (high confidence), MUC1 and MUC15 as immune-related extracellular-matrix mucins (high confidence), TLR2 as an innate immune sensor enriched in milk-EVs (high confidence), and HP as a host-defense acute-phase protein altered in mastitis-associated exosomes (medium confidence). The recurrence of this compact, functionally coherent set across all four species nominates it as a parsimonious cross-species inflammation signature of milk-EVs. Confidence levels in Table 1 were assigned from the strength of the originating annotation: “high” where the immune role is corroborated by multiple independent milk-EV studies and “medium” where it rests on a single or context-specific report, with supporting references listed for each marker in Table 1. As a sensitivity analysis, this source-annotated inflammation core was cross-checked against independent immune gene sets (see Section 4.4).

This signature is consistent with independent literature. Comparative analyses of cow, sheep, goat and buffalo milk EVs have reported enrichment of immune-modulating proteins as a shared, rather than species specific, property [17]; MUC1 is an established protease-resistant milk-EV surface glycoprotein; lactoferrin is a widely reported immunomodulatory milk-EV constituent; and a recent human breast-milk exosome study independently identified TLR2, alongside ICAM1 and FN1, as one of the principal proteins linking milk-EVs to pro-inflammatory and anti-infective responses [14]. Four of the five markers, therefore, have direct external corroboration, while HP and MUC15 are consistent with, though less frequently singled out in, the comparative literature.

### 2.4. Inflammation-Associated Evidence Across Molecular Classes

Beyond the conserved proteins, explicit inflammation annotation spanned multiple molecular classes. Of 20,556 evidence records, 905 (4.4%) carried a positive inflammation flag; these correspond to 772 distinct features, distributed across pathways (411), proteins (85), metabolites (84), study-level evidence (54), microRNAs (50), genes (44), datasets (27) and curated key entities (18) (Supplementary Table S2). This distribution reflects MetaMilkDB’s provenance model, in which the same molecular feature can accrue evidence from curated extraction, database queries and the primary literature; aggregate evidence was sourced 38% from curated extraction, 36% from database queries, and 26% from primary literature and publications. Notably, the inflammation signal was concentrated in pathway-level KEGG/GO annotations and in a focused set of proteins and microRNAs, rather than being diffusely distributed, supporting the biological specificity of the conserved inflammation core. The full inventory of these 773 features, with the species in which each was detected, is provided in Supplementary Table S2. Of these inflammation-annotated features, only the five-protein core (HP, LTF, MUC1, MUC15, TLR2) was shared across all four species, whereas the remaining inflammation-annotated proteins, microRNAs, metabolites and genes were predominantly species specific.

### 2.5. Inflammation-Annotated Pathways Converge on TLR/NF-κB Signaling

**Figure 2:**
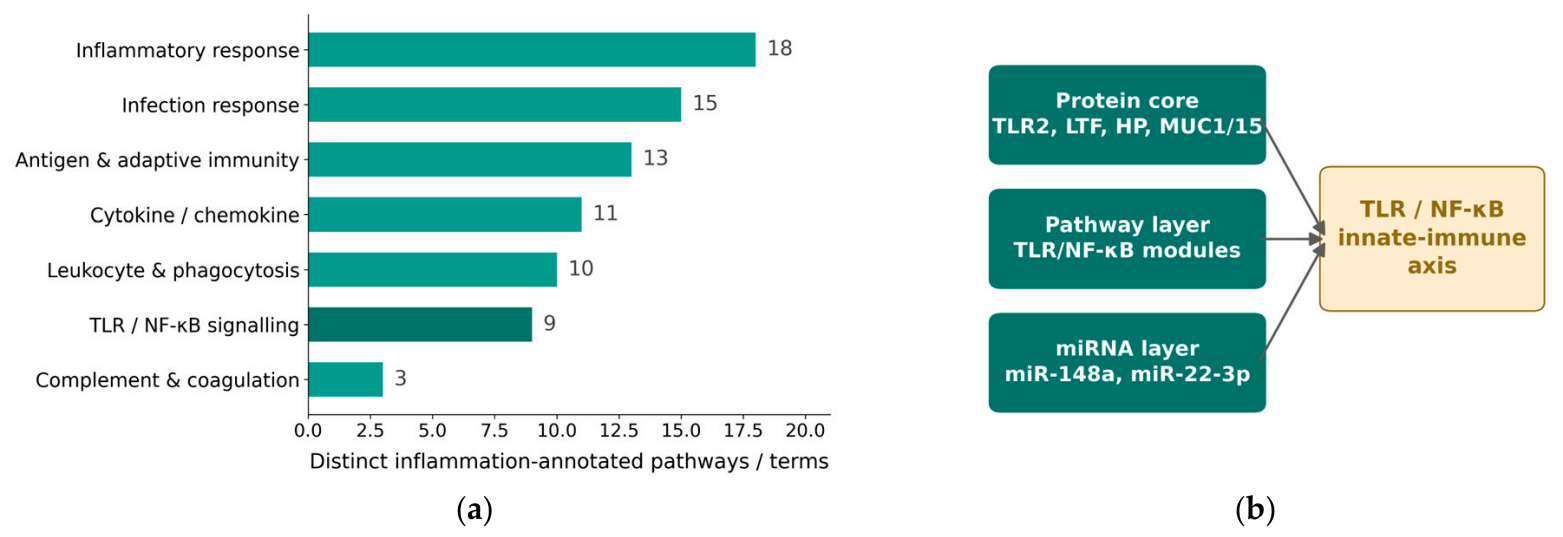
Inflammation-annotated pathway landscape of milk-EV cargo. (**a**) Distinct inflammation-flagged pathways and gene-ontology terms grouped into immune modules; the Toll-like-receptor/NF-κB module (highlighted) anchors the signature. (**b**) Convergence of the three conserved layers—protein core, inflammation-enriched pathways and gene-regulatory microRNAs—on a single TLR/NF-κB innate-immune axis.

To move from individual molecules to the signaling context in which they act, we examined the inflammation-flagged pathway and gene-ontology evidence. Of the 411 pathway/term records carrying an explicit inflammation flag, 249 were derived from KEGG and 119 from Gene Ontology source annotations, and 375 corresponded to distinct pathways or terms. Grouping the immune-related members thematically revealed a structured rather than diffuse landscape (Figure 2a): the largest modules were general inflammatory response (18 distinct terms), infection response (15), antigen processing and adaptive immunity (13), cytokine/chemokine signaling (11) and leukocyte function/phagocytosis (10), with a compact complement and coagulation module (3).

Most informative was the Toll-like-receptor/NF-κB signaling module (9 distinct pathways), which recurred across source studies and included the Toll-like receptor signaling pathway, its MyD88-dependent, MyD88-independent and TRIF-dependent branches, the NF-κB signaling pathway, and—strikingly—the TLR1:TLR2 and TLR6:TLR2 heterodimer signaling pathways. This module is mechanistically central to our findings: it is the pathway context of the conserved protein marker TLR2, and it is the same TLR4/NF-κB axis on which the conserved anti-inflammatory microRNAs act (Section 2.7). The pathway layer, therefore, provides an independent, annotation-level line of evidence that the conserved milk-EV inflammation program is organized around TLR-family innate-immune sensing rather than scattered across unrelated immune processes (Figure 2b).

### 2.6. Species-Specific Cargo Concentrates Adaptive-Immune Proteins, Most Strongly in Human Milk

**Figure 3:**
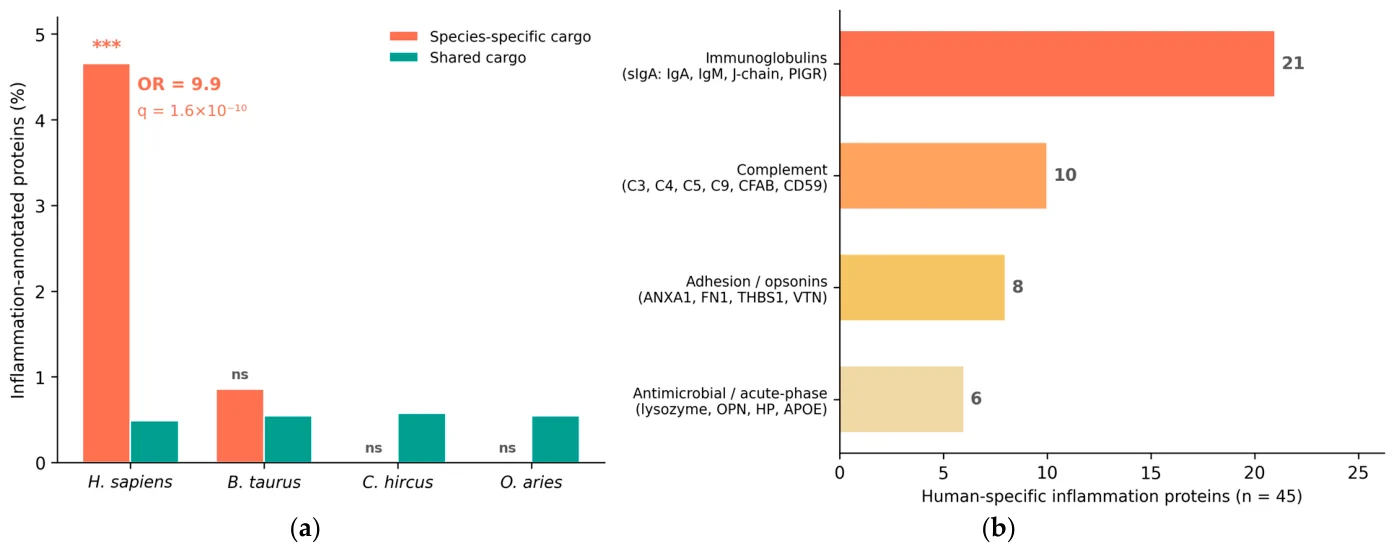
Species-specific concentration of inflammation cargo. (**a**) Percentage of inflammation-annotated proteins among species-specific versus shared milk-EV cargo, per species; human-specific cargo is strongly enriched (Fisher exact, Benjamini–Hochberg-corrected), with no equivalent signal in the ruminants. (**b**) Functional grouping of the 45 human-specific inflammation proteins, which form a coherent secretory-immunoglobulin and complement module. Statistical significance was assessed by one-sided Fisher exact test with Benjamini–Hochberg correction; ns, not significant (q ≥ 0.05); *** q < 0.001. Ig, immunoglobulin; J chain, joining chain; PIGR, polymeric immunoglobulin receptor.

Conservation describes only one axis of the data; the complementary question is which inflammation-related cargo is species specific rather than shared. We therefore tested, within each species, whether inflammation-annotated proteins were over-represented among proteins detected only in that species relative to those it shared with at least one other. The four species behaved strikingly differently. In human milk-EVs, inflammation-annotated proteins were strongly concentrated in the species-specific fraction: 45 of 966 human-specific proteins (4.7%) carried inflammation annotation, against only six of 1222 shared proteins (0.5%), a near ten-fold over-representation (Fisher exact odds ratio 9.9; *p* = 4 × 10^−11^; Benjamini–Hochberg q = 1.6 × 10^−10^). No equivalent enrichment was detected in bovine, caprine or ovine milk-EVs (q ≥ 0.49; Figure 3a). For comparison, inflammation-annotated proteins accounted for 8/931 (0.9%) of bovine-specific, 0/590 (0.0%) of caprine-specific and 0/557 (0.0%) of ovine-specific cargo, none of which reached significance; the species-specific inflammation proteins for each species are listed in Supplementary Table S3. In other words, inflammation-related cargo appears unremarkable when the conserved core is examined on its own (Section 2.3), but becomes strongly structured as soon as proteins are partitioned by species—and that structure is almost entirely confined to human milk.

The identity of the 45 human-specific inflammation proteins explains the asymmetry: they form a coherent adaptive- and humoral-immune module rather than a random scatter (Figure 3b). Twenty-one are components of the secretory-immunoglobulin system—immunoglobulin heavy and light chains, the joining (J) chain and the polymeric immunoglobulin receptor (PIGR) that transports secretory IgA into milk—ten belong to the complement cascade (C3, C4, C5, C9, factor B, C4b-binding protein, CD59, clusterin, C1-inhibitor), eight are adhesion or opsonising factors, and six are antimicrobial or acute-phase proteins. This composition recapitulates a well-established interspecies difference: the human-milk immunoglobulin repertoire is dominated by secretory IgA, whereas ruminant milk is dominated by IgG, which is transferred largely through different routes [18,19]. The conserved core and the divergent fraction, therefore, carry different kinds of immunity: the core is an innate, broadly shared scaffold (TLR2, lactoferrin, mucins), while the species-specific fraction—pronounced in human milk—is enriched for adaptive and complement-mediated effectors.

### 2.7. A Conserved EV-microRNA Layer Links the Signature to Gene Regulation

**Figure 4:**
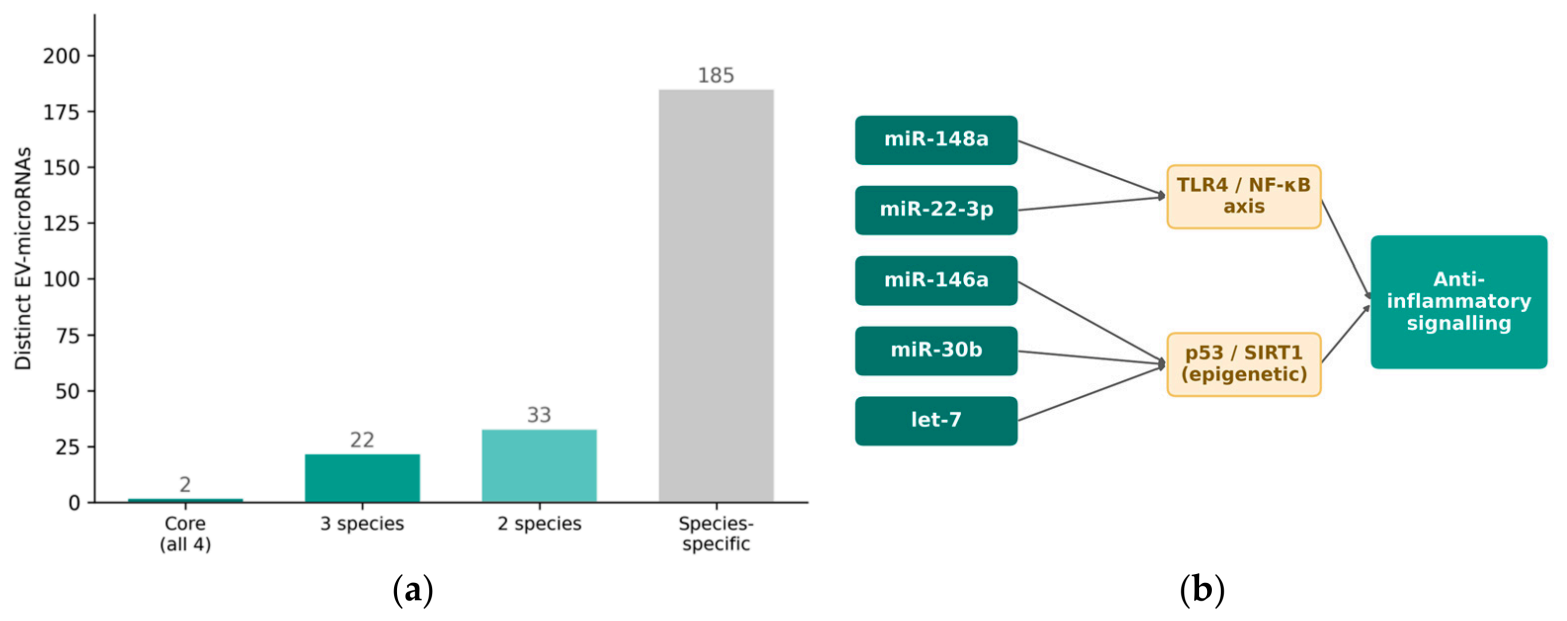
Conserved epigenetic EV-microRNA layer. (**a**) Partition of 242 distinct milk-EV microRNAs by the number of species in which each is detected; 24 are shared by ≥3 species. (**b**) Conserved, inflammation-annotated EV-microRNAs and their experimentally reported targets, converging on the TLR4/NF-κB innate-immune axis and on epigenetic regulators (p53/SIRT1) to produce net anti-inflammatory signaling.

The special focus of milk-EV biology on epigenetic, post-transcriptional gene regulation prompted us to examine the vesicle microRNA cargo alongside the protein signature. Grouping EV-microRNAs case-insensitively by mature-sequence identity after removing species prefixes yielded 242 distinct microRNAs across the four species, of which 24 were conserved in three or more species and two (miR-22-3p and miR-30a-5p) in all four (Figure 4a). A focused set of 44 EV-microRNAs carried explicit inflammation- or immune-related annotation, mirroring at the epigenetic level the compactness of the five-protein signature. The 44 inflammation-annotated EV-microRNAs, together with their per-species detection and conservation across one to four species, are listed in Supplementary Table S3.

Crucially, the conserved, inflammation-annotated microRNAs converge mechanistically on the same innate-immune axis as the conserved protein core. Several—most prominently miR-148a, the single most abundant microRNA of human milk-EVs—are annotated in source studies as anti-inflammatory regulators acting through the TLR4/NF-κB pathway, with experimentally validated targets (e.g., TLR4 and TRAF1 by luciferase assay in intestinal epithelial cells) [20]; miR-148a additionally targets the epigenetic regulators p53 and Sirtuin 1 [21,22], while the four-species-conserved miR-22-3p dampens inflammatory signaling via MAPK14, and the all-four-species miR-30a-5p has been reported to act on NF-κB in intestinal epithelium [23] (Figure 4b). Thus, the same TLR/NF-κB innate-immune module that defines the conserved protein signature (anchored by TLR2) is independently targeted, in the opposite regulatory direction, by a conserved set of EV-microRNAs. This two-layer convergence—conserved immune proteins delivered together with conserved gene-regulatory microRNAs that tune the very same pathway—suggests a candidate genetic-and-epigenetic model for how milk-EVs might transmit calibrated immune information from mother to offspring, a hypothesis that remains to be tested with direct functional assays. It should be noted that these microRNA–target relationships derive from functional studies in porcine, murine and human cell-line systems; their presence in milk-EVs of the four target species is inferred from mature-sequence identity and was not re-validated experimentally here.

### 2.8. In-House EV Proteomics Corroborate the Inflammation Signature

To test the conserved inflammation signature against data we generated ourselves, we examined the project’s in-house EV proteomes independently of the curated public layer. Across 18 in-house human milk-EV preparations, vesicle RNA yield (Qubit) ranged from 2.5 to 92.0 ng/µL (mean 28.5) and EV protein concentration from 0.33 to 3.13 µg/µL (mean 1.08), confirming recovery of intact, cargo-bearing vesicles suitable for proteomic profiling. Vesicle identity and purity of the in-house preparations were established following MISEV guidelines [24,25] and according to a previously published study [26]. At the proteome level, the in-house datasets contributed 265,009 protein measurements and recovered 4440, 711 and 982 distinct gene-level products in human, bovine and caprine milk-EVs, respectively.

Critically, four of the five signature markers—LTF, MUC1, MUC15 and TLR2—were independently re-detected in the in-house EV proteomes of all three profiled species (human, bovine and caprine), and HP in human and bovine preparations. Because these detections come from samples and instruments entirely separate from the curated literature on which the signature was originally defined, they provide internal, non-circular corroboration that the inflammation signature is a reproducible feature of milk-EV cargo rather than an artifact of any single published dataset.

## 3. Discussion

The principal observation of this analysis is the convergence of three molecular layers of the milk-EV cargo—proteins, enriched signaling pathways and microRNAs—on a single, evolutionarily conserved axis of innate immunity. Of 4958 EV protein families recovered across the four species, only 570 (11.5%) are common to all four, and within this conserved core, five proteins (HP, LTF, MUC1, MUC15 and TLR2) carry source-explicit inflammation annotation. The restriction of the inflammation signal to a compact and functionally related set, rather than to a representative cross-section of immune proteins, is consistent with selective constraint acting on the innate-immune component of milk-EV signaling across host lineages separated by tens of millions of years [11].

The functional annotations of the five markers indicate complementary, rather than redundant, roles. Lactoferrin (LTF) is an iron-sequestering antimicrobial that also modulates pro-inflammatory cytokine output; haptoglobin (HP), an acute-phase protein reported to be altered in mastitis-associated milk exosomes, reflects the inflammatory state of the mammary gland [19]. The mucins MUC1 and MUC15 contribute to the protective glycocalyx, limiting pathogen adhesion at the intestinal epithelium. MUC1 has additionally been described as a protease-resistant EV surface glycoprotein consistent with vesicle survival through gastrointestinal transit [17], whereas MUC15, a heavily glycosylated membrane-associated mucin, is less characterized but has been implicated in epithelial protection and cell adhesion [27]. TLR2 is a pattern-recognition receptor for bacterial cell-wall components, acting through TLR1:TLR2 and TLR6:TLR2 heterodimers [14]. Considered together, the set spans pathogen sensing, epithelial barrier maintenance, antimicrobial activity and the acute-phase response—effector functions relevant to the immature neonatal intestine, which depends on maternally supplied immune factors [1,11].

Pathway-level evidence is concordant with this interpretation. The inflammation-annotated pathway and gene-ontology terms are not uniformly distributed but cluster into a limited number of immune modules, among which a Toll-like-receptor/NF-κB module is prominent and includes the canonical TLR pathway and its MyD88-dependent, MyD88-independent and TRIF-dependent branches. This module provides the signaling context of the conserved marker TLR2 and links the protein layer to a defined innate-immune program rather than to dispersed immune processes.

The microRNA layer engages the same axis in the opposite regulatory direction. Several conserved, inflammation-annotated EV-microRNAs target components of TLR/NF-κB signaling: miR-148a, the most abundant microRNA of human milk-EVs, has been reported to attenuate TLR4/NF-κB signaling through validated targeting of TLR4 and TRAF1 in intestinal epithelial cells and to act on the regulators p53 and Sirtuin 1, while the four-species-conserved miR-22-3p has been shown to reduce inflammatory signaling via MAPK14 [21,22]. The co-packaging of immune effector proteins with microRNAs that constrain the same pathway is compatible with a self-limiting model of milk-EV immune signaling, in which activation is paired with negative regulation. Such a configuration would be biologically appropriate for the neonatal gut, which must respond to pathogens while tolerating dietary and commensal antigens encountered at the onset of enteral feeding, and its conservation across the four species is consistent with a function maintained by selection rather than specific to any single host [4].

Conservation of cargo identity does not, however, imply conservation of downstream function. Human milk-EVs expose tissue factor and promote coagulation, an activity absent from bovine milk-EVs, indicating that a shared molecular constituent can be deployed differently between hosts. The conserved set is therefore most appropriately regarded as a prioritized, mechanism-anchored core—the cargo fraction most likely to be functionally relevant and reproducibly detected across species—rather than as evidence of interchangeable biology. A further consideration is detection depth: because conservation is scored as presence across species, it is sensitive to how deeply each proteome was sampled, and the bovine arm is both the most studied and the deepest. As shown in the Results (Section 2.2), rarefying all species to the shallowest proteome preserved both a conserved fraction and the five-marker enrichment, indicating the core is robust to sampling depth even though its absolute size is best interpreted as an upper estimate. To assess this, all four species were down-sampled to the depth of the shallowest proteome (1838 families) and the analysis repeated; the four-species core decreased from 570 to a mean of 319 families (56%; 95% interval 299–335 over 200 iterations), and each of the five inflammation markers was retained in the rarefied core in 52–59% of iterations (Supplementary Figure S1). A conserved fraction and a recurrent enrichment of the five markers, therefore, persist after equalizing sampling effort, although the absolute size of the core is depth-dependent and is best interpreted as an upper estimate. The same caution applies to the species-divergence result: human milk is the most deeply sampled arm, which inflates the count of detectable species-specific proteins, including immunoglobulin variable domains. The near ten-fold enrichment is, however, far larger than the roughly two-fold difference in sampling depth between humans and the ruminants, and the secretory-IgA-over-IgG asymmetry it reflects is independently established, so the direction and broad magnitude of the effect are unlikely to be artifactual even if the precise odds ratio is depth-sensitive.

A second, and arguably more informative, axis emerges when the same inflammation annotation is partitioned by species rather than by conservation. The conserved core is not statistically enriched for inflammation relative to the rest of the EV proteome (5 of 570 conserved-core versus 57 of 4388 non-core proteins; Fisher exact OR = 0.67, *p* = 0.546, 95% CI 0.30–1.76); the inflammation signal instead concentrates in the species-specific fraction, and almost entirely in human milk, where inflammation-annotated proteins are nearly ten-fold over-represented among human-specific cargo. The molecules responsible—secretory immunoglobulins, the J chain and PIGR, and complement components—are adaptive and humoral effectors, in contrast to the innate sensing-and-barrier character of the conserved core. This dissociation is biologically coherent. Innate defense is ancient and broadly required, so the proteins that implement it (TLR2, lactoferrin, mucins) are shared and conserved; adaptive humoral immunity is comparatively plastic and is shaped by each species’ reproductive biology, and the dominance of secretory IgA in human milk—as opposed to IgG in ruminants—is a well-documented interspecies difference that our EV-level data recover de novo. The conserved core and the divergent fraction are therefore not competing findings but two faces of the same system: a common innate scaffold onto which each species layers its own adaptive complement. A practical corollary is that the conserved core is the rational basis for cross-species biomarkers and for animal-milk-EV applications, whereas the human-specific adaptive fraction is precisely the component that animal milk-EVs cannot supply and that distinguishes human milk functionally.

Two applications follow from these results. As a biomarker resource, a compact protein panel recurring across human and dairy species offers a cross-species readout of mammary and intestinal inflammation; HP is already associated with mastitis, and a panel validated in both human and animal milk could support comparable diagnostic assays across lactation contexts. For nutritional and therapeutic use, the conserved core represents the cargo fraction least likely to provoke xeno-incompatibility, providing a rationale for the use of bovine, caprine and ovine milk-EVs in human applications, including infant-formula supplementation and EV-based delivery [28]; the conserved anti-inflammatory microRNAs are candidate effectors in inflammatory intestinal conditions such as necrotising enterocolitis, in which milk-EV preparations have shown protective effects in model systems [10,21,22].

Taken together, the data support a view of milk as a vehicle for epigenetically active immune information transmitted from mother to offspring, with the conserved protein–pathway–microRNA module identified here as a candidate substrate for that transmission. Characterizing how the quantitative composition of this module varies with lactation stage, health status and species represents a logical next step toward defining its physiological role and assessing its translational potential [4,11].

Several limitations should be emphasized. The functional interpretations drawn herein, particularly the proposed self-limiting TLR/NF-κB circuit and the notion of maternal-to-infant immune transfer, rest on annotation-level and sequence-level evidence rather than direct functional assays, and the microRNA–target relationships derive from heterologous cell systems that were not re-validated in the four target species. Conservation and species specificity are scored as detection across curated datasets and therefore remain sensitive to differences in EV-isolation method, proteome depth and reporting completeness between source studies. In particular, EV-isolation methodology varied across the curated studies and can substantially influence the recovered proteome—especially the loosely associated EV surface (soft) corona—because milk-EV purification frequently relies on relatively harsh casein-depletion steps; we therefore record the isolation method reported by each study (Supplementary Table S1) and interpret surface-protein differences with corresponding caution. The conserved core and the human-specific adaptive fraction are accordingly best regarded as prioritized, hypothesis-generating cargo sets whose physiological roles require experimental validation.

## 4. Materials and Methods

### 4.1. Database Construction and Scope

MetaMilkDB is a relational database (SQLite (version 3.51.2) for development; PostgreSQL 16 (version 16.12) in production) integrating proteomic, transcriptomic, metabolomic, milk-composition, EV-characterization, and pathway-annotation evidence. Four target species were defined a priori—Homo sapiens (NCBI taxid 9606), Bos taurus (9913), *Capra hircus* (9925) and *Ovis aries* (9940). Two non-target species present in the source data (*Bubalus bubalis*, *Equus asinus*) were retained but flagged and excluded from conserved-core analyses. Evidence was drawn from 78 curated public studies [29,30,31,32,33,34,35,36,37,38,39,40,41,42,43,44,45,46,47,48,49,50,51,52,53,54,55,56,57,58,59,60,61,62,63,64,65,66,67,68,69,70,71,72,73,74,75,76,77,78,79,80,81,82,83,84,85,86,87,88,89,90,91,92,93,94,95,96,97,98,99,100] and from in-house project proteomics, organized into feature, annotation, evidence, identifier and study tables, each record carrying its source layer and study of origin. Inclusion was not restricted to a single EV-isolation strategy; instead, the EV-isolation method reported by each source study was recorded as metadata (summarized in Supplementary Table S1) so that its influence on downstream molecular composition could be considered during interpretation.

### 4.2. In-House EV Proteomics and Characterization

In addition to curated public studies, the project generated in-house milk-EV proteomics for human, bovine and caprine milk, recorded in MetaMilkDB under data sources flagged as in_house. All in-house human milk samples were collected with written informed consent from donors under a protocol approved by the Board of Directors of the University Research Institute for the Study and Treatment of Genetic and Malignant Diseases of Childhood (E.P.I.K.N.P.H.), National and Kapodistrian University of Athens (METAMILK research protocol), in accordance with the Declaration of Helsinki. EV isolation and characterization were performed and reported in accordance with MISEV guidelines [24,25]. EV preparations were characterized for particle-associated RNA (Qubit) and protein concentration; 18 human preparations with measured yields are summarized in the Results. In-house proteomic measurements (265,009 records) were ingested through the same identifier-resolution and provenance pipeline as the public layer and were analyzed separately when used to test reproducibility of the inflammation signature, so that in-house corroboration is independent of the curated evidence on which the signature was defined. In-house and public layers are distinguishable at all times via the source-layer field.

### 4.3. Identifier Resolution and Data-Honesty Policy

Features were resolved to canonical accessions (UniProt for proteins; standard gene and miRBase nomenclature for genes and microRNAs; HMDB/KEGG for metabolites). Where an identifier could not be confidently resolved, the feature was retained with an explicit needs-lookup status rather than being fabricated or removed. microRNA precursor and mature forms were merged case-insensitively with a transparent naming note. Entrez GeneID and UniProt accessions were not auto-merged across namespaces and were tagged accordingly. Critically, inflammation and immune annotations were assigned only where the originating study explicitly reported them; no inflammatory role was inferred from function, pathway membership or homology, and computational target predictions and de novo enrichment terms were excluded from evidence.

### 4.4. Conservation and Inflammation-Signature Analysis

EV-proteome conservation was computed over EV-compartment protein features from the four core species, grouped case-insensitively by canonical protein-family name. For each family, the set of species in which it was observed was determined, and families were partitioned by species count (1–4). The conserved core comprised families present in all four species. The inflammation core was defined as conserved-core families for which at least one evidence record carried a positive, source-explicit inflammation flag; each was reviewed for its annotated role and confidence. EV-microRNA conservation was computed analogously over RNA-type features, after normalizing microRNA names case-insensitively and stripping species prefixes (e.g., bta-, hsa-) so that orthologous mature microRNAs were grouped across species; microRNA target and pathway annotations were taken only where source studies reported them experimentally, and no targets were predicted de novo. For the species-divergence analysis, proteins detected in only one species were classified as species specific and the remainder as shared; within each species, over-representation of inflammation-annotated proteins among species specific versus shared cargo was tested by a one-sided Fisher exact test, and the four per-species *p*-values were corrected by the Benjamini–Hochberg procedure. The same one-sided Fisher exact test was additionally applied to the conserved core versus the remaining EV proteome to test whether the core itself was enriched for inflammation annotation. Because detection depth differs between species, this test was interpreted alongside the rarefaction analysis and against the known immunoglobulin-class differences between human and ruminant milk. Inflammation-annotation pathway analysis used the curated pathway and gene-ontology features carrying a source-explicit inflammation flag; KEGG and GO terms were grouped into immune modules by controlled keyword matching, and counts reflect distinct source-annotated pathways without any de novo enrichment computation. As a sensitivity analysis, the source-annotated inflammation flag underlying the con-served core was cross-checked against three independent immune reference sets—GO immune-system-process terms, KEGG/Reactome immune pathways, and the UniProt keyword “Immunity”; four of the five markers (HP, LTF, MUC1 and TLR2) were recovered by at least one independent immune reference set (Gene Ontology, 4/5; KEGG/Reactome, 1/5; UniProt “Immunity”, 2/5), whereas MUC15—a less-characterized epithelial adhesion mucin—was not, indicating that the signature is largely reproducible under annotation schemes independent of our own curation.

## Figures and Tables

**Table 1:** The conserved five-protein inflammation core of milk-EVs.

Protein	Source-Annotated Role	Confidence	Functional Class
LTF	Antimicrobial (lactotransferrin/lactoferrin); pathogen protection	High	Innate defense
MUC1	Mucin; immune-related extracellular-matrix constituent	High	Barrier/mucin
MUC15	Mucin; immune-related extracellular-matrix constituent	High	Barrier/mucin
TLR2	Innate immune sensor; TLR2 signaling enriched in milk-EVs	High	Immune sensing
HP	Host-defense acute-phase protein altered in mastitis exosomes	Medium	Acute-phase

## Data Availability

The curated datasets underlying the figures reported in this study, together with the MetaMilkDB analysis outputs and curation rules, are available from the corresponding author on reasonable request. The in-house EV proteomic data were generated within earlier project work and are subject to consortium data-governance restrictions; they are flagged as in-house within MetaMilkDB. Supplementary Tables S1–S5 contain the curated data underlying the conclusions.

## Supplementary Materials

The following supporting information can be downloaded at: https://www.mdpi.com/article/10.3390/ijms27188142/s1. References [29,30,31,32,33,34,35,36,37,38,39,40,41,42,43,44,45,46,47,48,49,50,51,52,53,54,55,56,57,58,59,60,61,62,63,64,65,66,67,68,69,70,71,72,73,74,75,76,77,78,79,80,81,82,83,84,85,86,87,88,89,90,91,92,93,94,95,96,97,98,99,100] are cited in the Supplementary Materials.

## Abbreviations

The following abbreviations are used in this manuscript:

EV	extracellular vesicle
milk-EV	milk-derived extracellular vesicle
HP	haptoglobin
LTF	lactotransferrin/lactoferrin
MUC1	mucin 1
MUC15	mucin 15
TLR2	Toll-like receptor 2
TLR4	Toll-like receptor 4
NF-κB	nuclear factor kappa B
MyD88	myeloid differentiation primary response 88
TRIF	TIR-domain-containing adapter-inducing interferon-β
TRAF1	TNF receptor-associated factor 1
IgA	immunoglobulin A
IgG	immunoglobulin G
PIGR	polymeric immunoglobulin receptor
J chain	joining chain
SIRT1	sirtuin 1
MAPK14	mitogen-activated protein kinase 14
miRNA	microRNA
KEGG	Kyoto Encyclopedia of Genes and Genomes
GO	Gene Ontology
HMDB	Human Metabolome Database
NTA	nanoparticle tracking analysis
TEM	transmission electron microscopy
MISEV	Minimal Information for Studies of Extracellular Vesicles
FDR	false discovery rate
OR	odds ratio
CI	confidence interval
NEC	necrotising enterocolitis
MFGM	milk-fat-globule membrane
